# Inter- and Intra-Individual Variation in Allele-Specific DNA Methylation and Gene Expression in Children Conceived using Assisted Reproductive Technology

**DOI:** 10.1371/journal.pgen.1001033

**Published:** 2010-07-22

**Authors:** Nahid Turan, Sunita Katari, Leigh F. Gerson, Raffi Chalian, Michael W. Foster, John P. Gaughan, Christos Coutifaris, Carmen Sapienza

**Affiliations:** 1 Fels Institute for Cancer Research and Molecular Biology, Temple University School of Medicine, Philadelphia, Pennsylvania, United States of America; 2 Department of Obstetrics and Gynecology, University of Pennsylvania School of Medicine, Philadelphia, Pennsylvania, United States of America; 3 Biostatistics Consulting Center, Temple University School of Medicine, Philadelphia, Pennsylvania, United States of America; 4 Department of Pathology and Laboratory Medicine, Temple University School of Medicine, Philadelphia, Pennsylvania, United States of America; Massachusetts General Hospital, Howard Hughes Medical Institute, United States of America

## Abstract

Epidemiological studies have reported a higher incidence of rare disorders involving imprinted genes among children conceived using assisted reproductive technology (ART), suggesting that ART procedures may be disruptive to imprinted gene methylation patterns. We examined intra- and inter-individual variation in DNA methylation at the differentially methylated regions (DMRs) of the *IGF2/H19* and *IGF2R* loci in a population of children conceived *in vitro* or *in vivo*. We found substantial variation in allele-specific methylation at both loci in both groups. Aberrant methylation of the maternal *IGF2*/*H19* DMR was more common in the *in vitro* group, and the overall variance was also significantly greater in the *in vitro* group. We estimated the number of trophoblast stem cells in each group based on approximation of the variance of the binomial distribution of *IGF2*/*H19* methylation ratios, as well as the distribution of X chromosome inactivation scores in placenta. Both of these independent measures indicated that placentas of the *in vitro* group were derived from fewer stem cells than the *in vivo* conceived group. Both IGF2 and H19 mRNAs were significantly lower in placenta from the *in vitro* group. Although average birth weight was lower in the *in vitro* group, we found no correlation between birth weight and IGF2 or IGF2R transcript levels or the ratio of IGF2/IGF2R transcript levels. Our results show that *in vitro* conception is associated with aberrant methylation patterns at the *IGF2*/*H19* locus. However, very little of the inter- or intra-individual variation in H19 or IGF2 mRNA levels can be explained by differences in maternal DMR DNA methylation, in contrast to the expectations of current transcriptional imprinting models. Extraembryonic tissues of embryos cultured *in vitro* appear to be derived from fewer trophoblast stem cells. It is possible that this developmental difference has an effect on placental and fetal growth.

## Author Summary

We have screened a population of children conceived *in vitro* for epigenetic alterations at two loci that carry parent-of-origin specific methylation marks. We made the observation that epigenetic variability was greater in extraembryonic tissues than embryonic tissues in both groups, as has also been demonstrated in the mouse. The greater level of intra-individual variation in extraembryonic tissues of the *in vitro* group appears to result from these embryos having fewer trophoblast stem cells. We also made the unexpected observation that variability in parental origin-dependent epigenetic marking was poorly correlated with gene expression. In fact, there is such a high level of inter-individual variation in IGF2 transcript level that the presumed half-fold reduction in IGF2 mRNA accounted for by proper transcriptional imprinting *versus* complete loss of imprinting would account for less than 5% of the total population variance. Given this level of variability in the expression of an imprinted gene, the presumed operation of “parental conflict” as the selective force acting to maintain imprinted gene expression at the *IGF2*/*H19* locus in the human should be revisited.

## Introduction

Several epidemiological studies have reported a higher incidence of rare disorders involving imprinted genes (Angelman syndrome [1]–[3] and Beckwith-Wiedemann syndrome [4]–[8]) among children conceived using assisted reproductive technologies (ART). Studies on imprinted gene expression and parental allele-specific DNA methylation in animal models have also suggested that epigenetic marks may be altered by treatments and procedures commonly employed in ART (ovarian stimulation, egg retrieval, *in vitro* fertilization, intracytoplasmic sperm injection, preimplantation embryo culture, embryo transfer) [9]–[13].

CpG sites in differentially methylated regions (DMRs) of imprinted genes are methylated on the allele contributed by one parent and unmethylated on the allele contributed by the other. This pattern of differential allelic methylation is established during male and female gametogenesis [14], [15] and the differences are maintained after fertilization such that cells from most somatic tissues are expected to exhibit the same parental allele-specific methylation pattern [16], [17]. However, if ART treatments and procedures in the human are disruptive to imprinted gene methylation patterns, as they are in the mouse [18], one might predict that alterations could occur in some cells of the early embryo but not in others, resulting in individuals who were mosaic to varying degrees for loss or relaxation of proper imprinted allelic methylation. In addition, different degrees of relaxation of allele-specific methylation could be observed between different tissues of the same individual, depending on when the disruption occurred during development.

One of the loci shown to be susceptible to alteration of epigenetic modifications by *in vitro* culture and ovarian stimulation in the mouse is *Igf2*/*H19* [11], [13], [19]–[23]. Because IGF2 is an important placental growth factor and one of the phenotypes most strongly associated with human ART procedures is low birth weight, we reasoned that the human *IGF2*/*H19* locus might also be susceptible during ART treatments and procedures. We compared parental allele-specific methylation between children conceived *in vitro* or *in vivo* at the DMR that functions as an imprint control region (ICR) at *IGF2*/*H19* [24], [25] and also at an *IGF2* receptor (*IGF2R*) DMR [26], [27]. We examined a sample of cord blood, a section of umbilical cord and five sections of placenta in each child for abnormal methylation of maternal alleles at the *IGF2*/*H19* ICR/DMR and for abnormal methylation of paternal alleles at the *IGF2R* DMR [28]. Under our null hypothesis, little variation in parental allele-specific methylation was expected within an individual, or between individuals, because the methylation status of each CpG site in the DMR is set in the gametes [29] and faithful replication of this status during development is expected to result in the same allelic methylation ratio in each individual and in each tissue. We also measured steady-state IGF2, H19 and IGF2R transcript levels to determine whether mRNA levels were correlated with abnormal allelic methylation ratio or birth weight. To our knowledge, this is the first study to examine intra-individual variation in epigenetic markings in children conceived using assisted reproduction.

## Results

### Intra- and Inter-Individual Variation in DNA Methylation at the *IGF2/H19* DMR

We investigated intra- and inter-individual variation in allele-specific methylation at the *IGF2/H19* DMR. We measured the relative level of CpG methylation on maternal and paternal alleles at this locus in cord blood, cord and five sections of placenta taken from a population of children conceived either *in vitro* or *in vivo*.

The imprinted *IGF2/H19* DMR is located between the *IGF2* and *H19* genes and is normally methylated on only the paternal allele [24], [30]. We used a single nucleotide polymorphism (a C/T SNP at a *CfoI* site) to identify informative (heterozygous) individuals and a methylation-sensitive restriction endonuclease (*MluI*) to determine the methylation status of a specific CpG site within the DMR, as described previously [28]. Methylation at the *MluI* site, and an adjacent *MaeII* site, have been shown previously to be characteristic of the methylation at surrounding CpGs by bisulfite sequencing (Fig. 7 in Sandovici et al., 2003) [28]. We identified 45 *in vitro* and 56 *in vivo* individuals who were informative and for whom DNA was available from cord blood, cord and five sections of placenta.

Because previous studies have indicated that loss or relaxation of imprinting is a quantitative trait [31], [32], we measured the ratio between DNA methylation levels on maternal and paternal (M/P) alleles as an indicator of imprinting status. A ratio of zero corresponds to exclusive methylation of the *MluI* site on the paternal allele, while a ratio of one signifies methylation of this site on an equal number of maternal and paternal alleles (*n.b*.: Although we have not determined the parental origin of each allele in the present study because DNA was not available from parents, we have shown previously that the less methylated allele was maternal in all 163 individuals for whom we were able to determine parental origin by pedigree analysis [28]. We therefore assume that the less methylated allele is maternal in the population examined here, also.) In the case of controls, no uncleaved C alleles were detected in any C/C homozygous individuals, indicating that *CfoI* cleaved the “hot-stop” PCR products with >99% efficiency [33].

**Figure 1:**
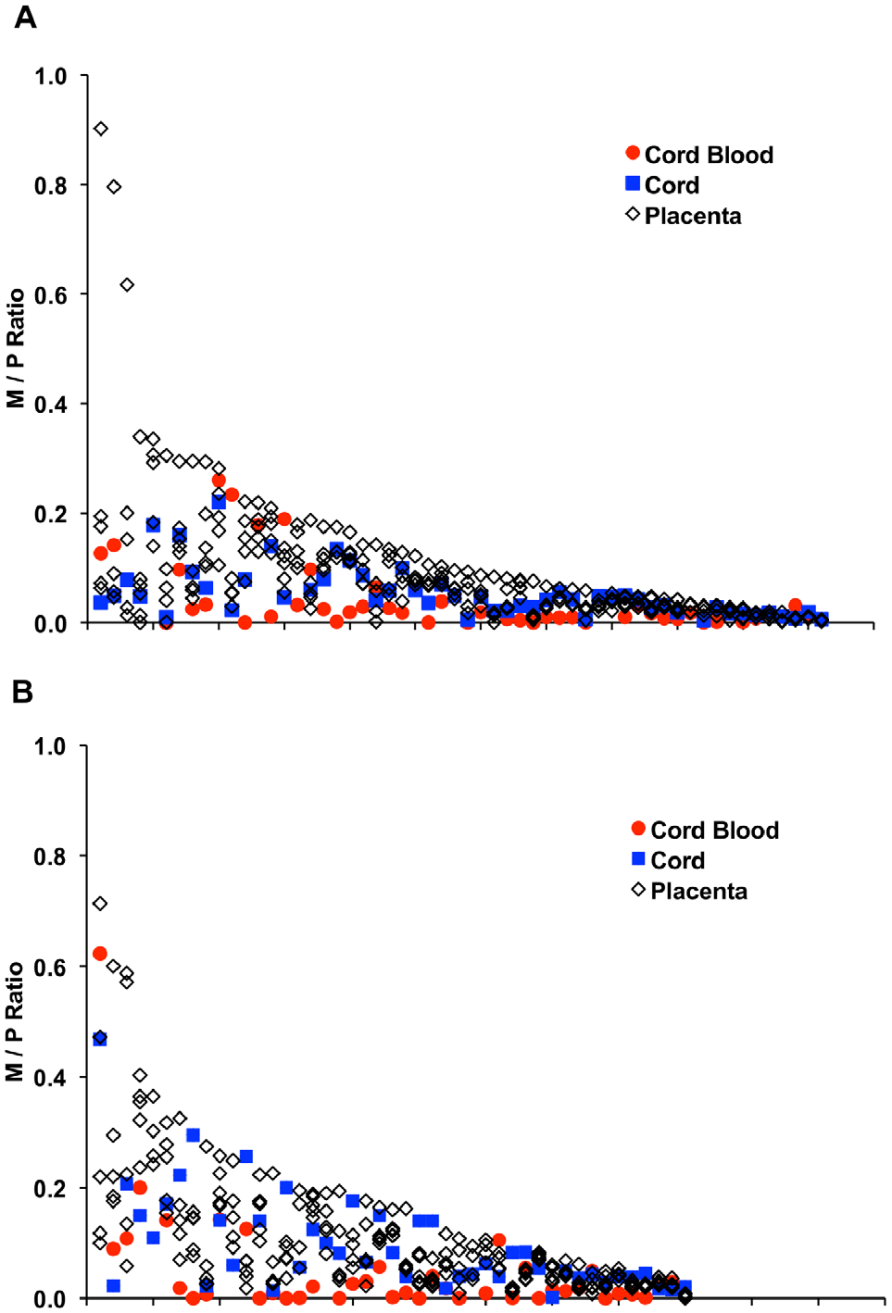
Allele-specific methylation at the *IGF2/H19* DMR. (A) 56 informative *in vivo* individuals, and (B) 45 informative *in vitro* individuals. M/P represents the DNA methylation ratio between maternal and paternal alleles (see text). Each individual is represented as vertical data-set of M/P ratios from cord blood, cord and five sections of placenta. Individuals were ranked based on the degree of scatter (intra-individual variation) observed.

Figure 1 shows the distribution of M/P methylation ratios observed from 56 informative *in vivo* (Figure 1A) and 45 informative *in vitro* (Figure 1B) individuals. The M/P ratios were measured for cord blood, cord and five sections of placenta from each informative individual. The data are represented as a series of symbols on a vertical line, ranked from individuals showing the greatest range of variation on the left side of the graph to individuals showing the least range of variation on the right side of the graph. The distribution of individual M/P methylation ratios in cord blood (red circles in Figure 1) shows that the great majority of informative individuals have <15% methylation on the maternal allele in both groups. Approximately 8% of the population examined here has ≥15% of methylation of CpG sites on the maternal allele in cord blood. These findings are similar to those reported previously by Sandovici et al. (2003) for M/P methylation ratios measured in peripheral blood samples from the CEPH families [28]. The distribution of individual M/P methylation ratios in cord showed a similar pattern to that observed in cord blood (Figure 1), while the five sections of placenta taken from each individual showed a broad range of intra-individual variation in M/P methylation ratios.

When individual tissues are compared, both M/P ratio mean and variance are greater in the *in vitro* group in each tissue, although two of these comparisons (cord blood means, in which the fewest samples are compared between the two groups, and placenta variance, in which the greatest range of variation is observed) do not reach statistical significance (Table 1). However, because the *a priori* expectation for the mean M/P methylation ratio of the *IGF2*/*H19* DMR is near zero, independent of the embryonic origin of the tissue (because the methylation status of maternal and paternal DMR alleles is assumed to be determined in the gametes and to escape the genome wide demethylation/remethylation that occurs in preimplantation embryos) [16], it is not inappropriate to combine all samples in each group to determine whether the two groups differ in mean M/P ratio and whether the groups have equal variance. When samples from all tissues are combined, both allele-specific methylation ratio mean and variance are significantly greater in the *in vitro* group (*P* = 0.0001 and *P* = 0.0006, respectively, Table 1). The fact that the *intra*-individual variation in M/P ratios is also greater in the *in vitro* group may be seen, simply, by comparing the fraction of individuals in each group in which all samples have M/P ratios below any arbitrarily chosen value. For example, only 31% of *in vitro* individuals (14/45) maintain M/P ratios of <0.1 in cord blood, cord and all five sections of placenta while 46% of *in vivo* individuals (26/56) are below this threshold.

**Table 1:** Intra- and inter-individual variation in DNA methylation at the *IGF2/H19* DMR.

Tissue	Group^a^	Mean	Variance	Means^b^ (*P*)	Variances (*P*)
Cord Blood	*In vitro*	0.0617	0.0135	0.2546	0.0001
	*In vivo*	0.0416	0.0041		
Cord	*In vitro*	0.0993	0.0083	0.0075	<0.0001
	*In vivo*	0.0550	0.0023		
Placenta	*In vitro*	0.1017	0.0111	0.0059	0.0620
	*In vivo*	0.0801	0.0091		
Total	*In vitro*	0.0970	0.0110	0.0001	0.0006
	*In vivo*	0.0716	0.0077		

^**a**^M/P methylation ratios measured in cord blood, cord, and five sections of placenta tissue from 45 informative *in vitro* individuals and 56 informative *in vivo* individuals.

^**b**^Wilcoxon Rank Sums Test (*P*≤0.05 considered significant).

### Greater Intra-Individual Variance in Placenta M/P Ratios Is Associated with Fewer Trophoblast Stem Cells

One mechanism by which greater variance in an epigenetic character may occur is through a sampling effect that depends on the number of stem cell progenitors that give rise to a particular tissue; the fewer the number of stem cells, the greater the variance. Because much of the difference in variance observed between the two groups occurs as a result of intra-individual differences in umbilical cord and placenta samples, we estimated the number of trophoblast stem cells that give rise to the placenta in each group by comparing the distribution of X-inactivation scores in females from each comparison group [34], [35] and by comparing the distribution of M/P *IGF2*/*H19* methylation ratios from Figure 1. We note that the assay used in each case amounts to a simple yes/no binomial trial of the form “is the CpG site being examined methylated (in which case it gives a signal) or not (in which case it does not)”, each of which is expected to yield a “success” (the DNA molecule in question is methylated) with probability “p” (p = 0.5 in the case of which X chromosome is inactivated and p = 0.1 in the case of methylation of the maternal *IGF2*/*H19* DMR, see below) or a failure with probability “q” (which is equal to 1-p).

The number of trophoblast stem cells may be estimated from the distribution of X-inactivation scores by comparing the actual distribution of X-inactivation scores with the distribution estimated from the variance of the binomial distribution (pq/N), setting the probability that either allele is methylated (p or q) to 0.5 and generating the distribution for different values of N (number of stem cells) [34], [35]. We determined the X-inactivation score distribution for each group (using DNA samples from five sections of placenta from 50 *in vitro* and 54 *in vivo* females) by comparing allele-specific methylation at *HpaII* sites adjacent to the CAG trinucleotide repeat in the highly polymorphic Androgen Receptor locus (*AR*) [36]. The closest fit to the distribution of X-inactivation scores in placenta in children conceived *in vitro* corresponds to nine trophoblast stem cells and the closest fit in children conceived *in vivo* corresponds to 11 trophoblast stem cells (Table 2).

**Table 2:** Determining number of stem cells by approximating the variance of binomial distribution of X-inactivation and M/P *IGF2/H19* methylation ratio results.

Assay	Tissue	Group	Mean	SD^c^ or Variance^d^	Number of stem cells
X-inactivation^a^	Placenta	*In vitro*	0.4775	0.1637^c^	9.33
		*In vivo*	0.4824	0.1502^c^	11.09
*IGF2/H19* DMR methylation^b^	Placenta	*In vitro*	0.1017	0.0111^d^	8.15
		*In vivo*	0.0801	0.0091^d^	9.90

^**a**^X-inactivation ratios were measured in five sections of placenta from 50 *in vitro* and 54 *in vivo* females using the HUMARA PCR assay.

^**b**^Allele-specific methylation ratios at the *IGF2/H19* DMR were measured in five sections of placenta from 45 informative *in vitro* individuals and 56 informative *in vivo* individuals.

We additionally estimated the number of trophoblast stem cells in each group by comparing the distribution of M/P *IGF2*/*H19* DMR methylation ratios (Figure 1) from the two comparison groups, using 0.1 and 0.9 as values for p and q (these values were selected based on the observation that ∼10% of individuals have significant methylation on the maternal DMR while ∼90% have very few cells carrying maternal DMR methylation [28]. These values are also in close agreement with the probability that any maternal DMR DNA molecule is methylated (placenta *in vivo* mean  = 0.0801, *in vitro* mean  = 0.1017, Table 1). Using these parameters, the closest fit to the distribution of M/P ratios in placenta in children conceived *in vitro* corresponds to eight trophoblast stem cells and the closest fit in children conceived *in vivo* corresponds to 10 trophoblast stem cells (Table 2).

Overall, these very similar independent estimates of between-group epigenetic variation (*n.b.*: not only are the two loci examined on different chromosomes but many of the individuals in the X-inactivation groups, composed of only females, and the *IGF2*/*H19* groups were different) are consistent with the prediction that overall greater variance in M/P *IGF2*/*H19* DMR methylation ratios in the *in vitro* group is associated with fewer trophoblast stem cells.

### Analyses of *IGF2*/*H19* DMR Methylation in Placenta by Pyrosequencing

**Figure 2:**
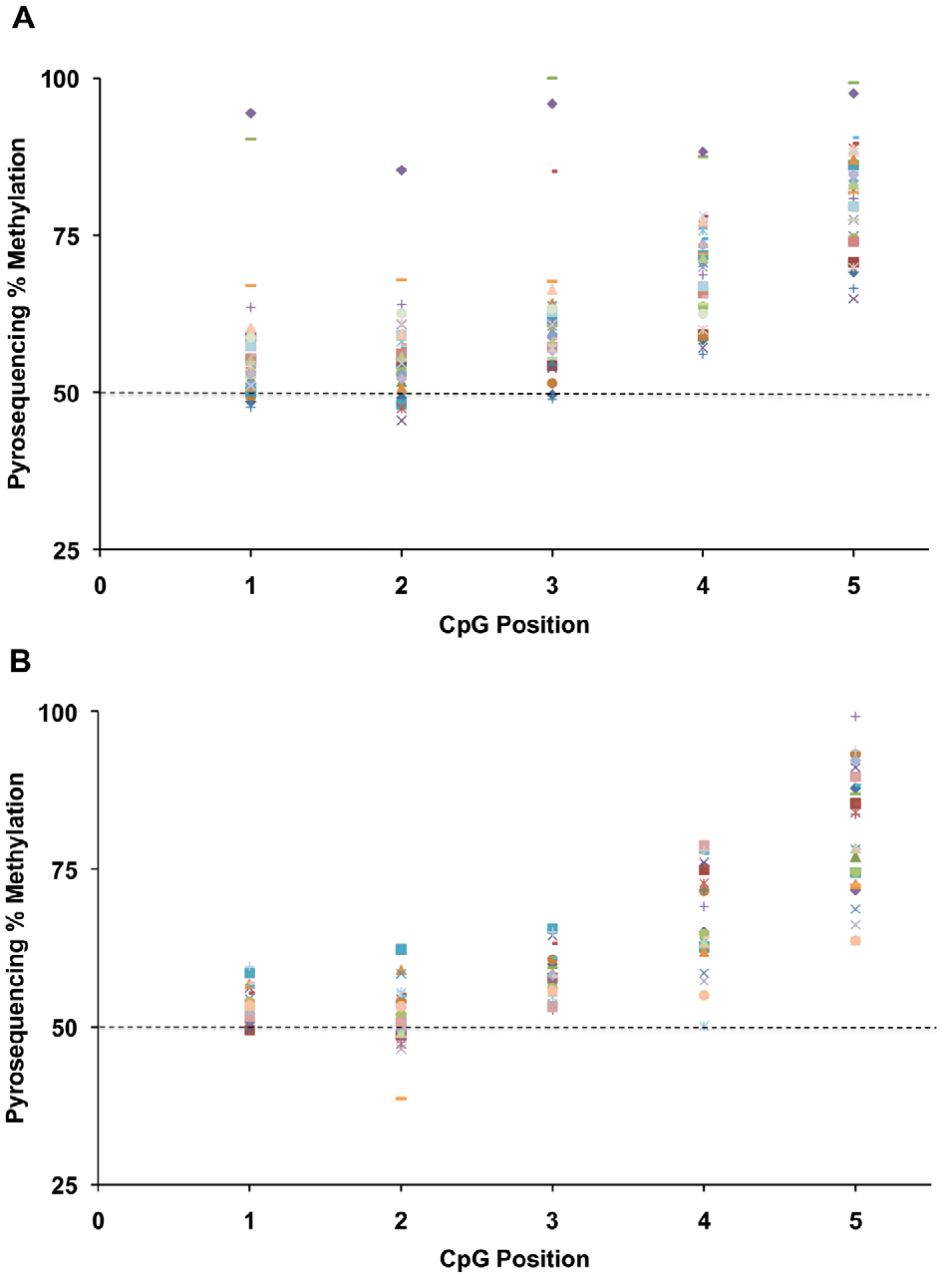
Pyrosequencing methylation at the *IGF2/H19* DMR in placenta. (A) 31 *in vivo* individuals, and (B) 26 *in vitro* individuals. The methylation results at each CpG site for each individual are represented by a horizontal series of symbols. All but a few of the CpGs show methylation levels of 50% or higher, which is in concordance with our findings of gain of methylation on the maternal allele (M/P ≥0.15 in placenta, Figure 1).

Because the methylation-sensitive restriction endonuclease assay used to generate the data shown in Figure 1 provides a ratio of maternal alleles at which *MluI* sites are methylated to paternal alleles at which *MluI* sites are methylated rather than an absolute fraction of all alleles, we also assayed DNA methylation at the *IGF2/H19* DMR by bisulfite pyrosequencing. The assay we used quantifies the methylation status of five CpGs in the *IGF2/H19* DMR that are adjacent to the CpG queried in the *MluI* assay. If an M/P ratio greater than zero (Figure 1) represents methylation of maternal alleles in addition to methylation of DMRs on all paternal alleles, then the CpG sites in these samples/individuals should be methylated on greater than 50% of molecules assayed by bisulfite pyrosequencing (*i.e*. all paternal alleles plus some fraction of maternal alleles). The placenta samples with M/P ratios greater than zero show greater than 50% methylation at all five CpGs in almost all cases (Figure 2) by bisulfite pyrosequencing, indicating that both the methylation-sensitive restriction endonuclease assay and the bisulfite pyrosequencing assay are measuring gain of methylation on maternal alleles, as has also been reported in the mouse [19], [22], [23].

### Allele-Specific Expression of *IGF2*/*H19* Is Not Disrupted by Bi-Allelic Methylation of the DMR

The current model for imprinted transcriptional control of *IGF2/H19* correlates methylation at the ICR/DMR with an inability to bind CTCF, transcriptional silencing of *H19* and transcriptional activation of *IGF2* [37], [38]. Because our analysis of DMR methylation indicates that all paternal and some maternal DMRs are methylated, we examined mRNA expression in individuals with varying levels of bi-allelic DMR methylation, for the presence of bi-allelic transcripts of IGF2 and H19. We assayed individuals who were informative for an *ApaI* polymorphism (in exon 9) for the presence of transcripts from both *IGF2* alleles [32]. We also assayed individuals who were informative for an *RsaI* polymorphism (in exon 5) for the presence of transcripts from both *H19* alleles [39], which would be expected to occur if paternal DMRs became demethylated.

Although we detected minor amounts of presumed maternal IGF2 mRNA in several individuals, there was no correlation between maternal DMR methylation and amount of transcript from the maternal allele (Table 3). We also observed only small amounts of presumed paternal allele expression of H19 mRNA, which suggested that there was no loss of methylation on paternal alleles in these samples (Table 3), also consistent with the overall greater than 50% methylation observed by pyrosequencing (Figure 2).

**Table 3:** Allelic-specific expression of IGF2 and H19 in human placenta.

Tissue	Group	Maternal/Paternal Ratio^a^	IGF2 Maternal Expression^b^	H19 Paternal Expression
Placenta	*In vitro*	0.016	2.9%	
		0.020	6.9%	
		0.023	7.0%	
		0.027	1.6%	
		0.032	15.8%	
		0.055		2.5%
		0.058		1.9%
		0.068		1.2%
		0.156		1.6%
		0.184		1.0%
		0.472		0.8%
	*In vivo*	0.004	2.8%	
		0.027	2.6%	
		0.088		3.0%
		0.187		2.1%
		0.236	5.5%	
		0.307	8.8%	

^**a**^DNA methylation levels on maternal and paternal (M/P) alleles (a ratio of zero corresponds to exclusive methylation of the *MluI* site on the paternal allele, while a ratio of one signifies methylation of this site on an equal number of maternal and paternal alleles).

^**b**^The threshold for scoring loss of imprinting for IGF2 was a ratio of less than 3∶1 between the more-abundant and less abundant alleles [32].

### Intra-Individual Variation in DNA Methylation at the *IGF2R* DMR

We investigated intra-individual variation in allele-specific methylation at an *IGF2R* DMR we have also examined previously [28]. The rationale for examining allele-specific methylation at this non-transcriptionally imprinted locus is that this locus may be a more sensitive reporter of any disruption in CpG site methylation by environmental factors because such changes are not predicted to affect transcription and are less likely to be selected against. The relative level of CpG methylation on paternal and maternal alleles at this locus was measured in cord blood, cord and five sections of placenta taken from populations of children conceived either *in vitro* or *in vivo.* The less methylated allele is assumed to be paternal because we found no individuals in which the paternal allele was more methylated than the maternal allele in 112 informative individuals for whom allelic inheritance could be confirmed by pedigree analysis [28].

The parental origin-specifically methylated *IGF2R* DMR is located in the second intron of *IGF2R* and is normally methylated on the maternal allele. Although the human *IGF2R* gene is transcribed from both alleles [40] differential methylation of maternal and paternal alleles is maintained in the human, as it is in the mouse [26] and a small fraction of the human population may have transcriptional imprinting of *IGF2R* [27]. We used a single nucleotide polymorphism within an *MspI* site to identify maternal and paternal alleles of informative (heterozygous) individuals and a methylation-sensitive restriction endonuclease (*NotI*) to determine the methylation status of a specific CpG site within the DMR [28]. We identified 28 *in vitro* and 27 *in vivo* individuals who were informative and assayed allele-specific methylation as described previously [28].

We calculated the ratio between the DNA methylation levels on paternal and maternal (P/M) alleles as an indicator of methylation imprint status. A ratio of zero corresponds to exclusive methylation of the *NotI* site on the maternal allele, while a ratio of one signifies methylation of this site on an equal number of paternal and maternal alleles. In the case of controls, no uncleaved C alleles were detected in any C/C homozygous individuals, indicating that *MspI* cleaved the PCR products with >99% efficiency.

**Figure 3:**
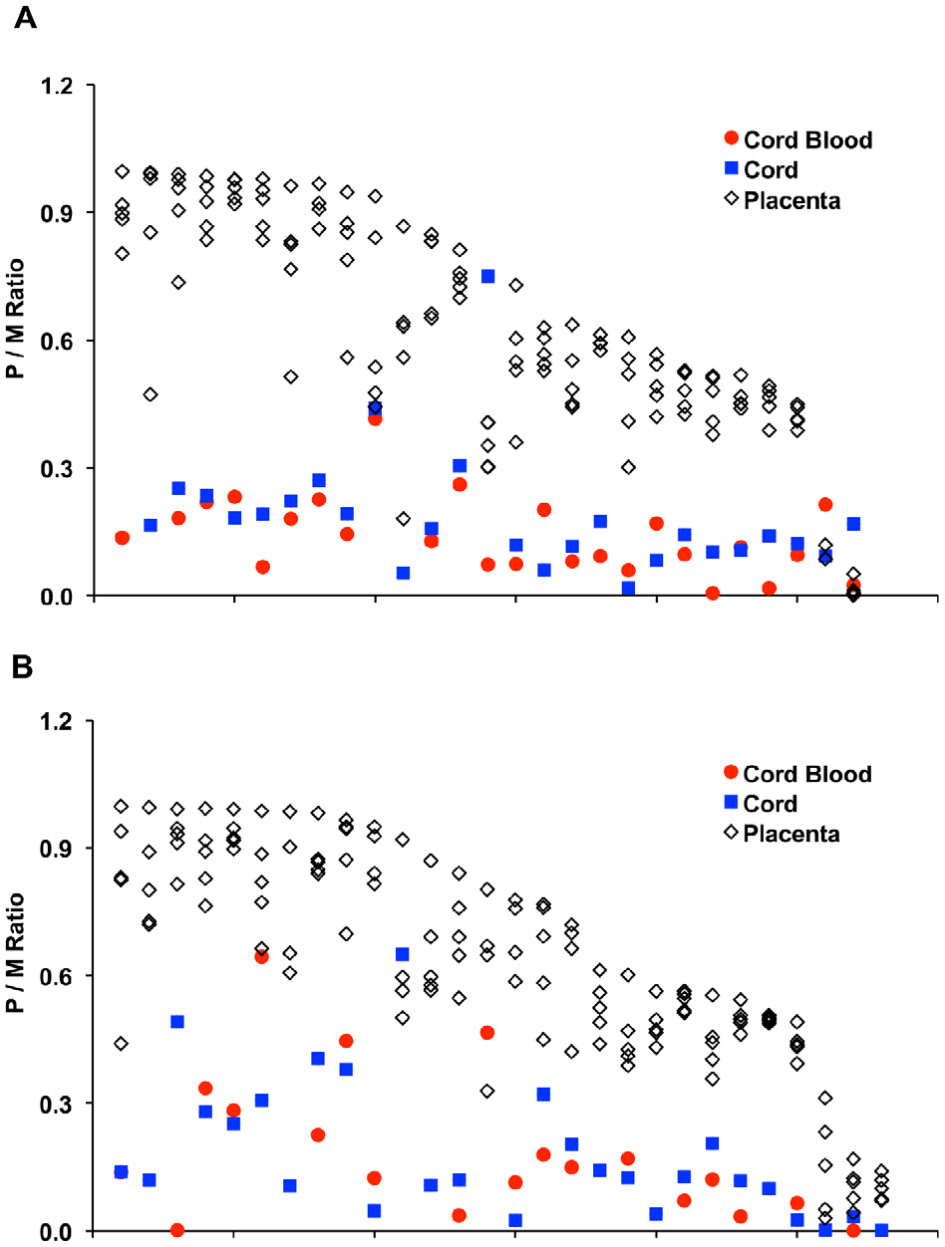
Allele-specific methylation at the *IGF2R* DMR. (A) 27 informative *in vivo* individuals, and (B) 28 informative *in vitro* individuals. P/M represents the DNA methylation ratio between paternal and maternal alleles (see text). Each individual is represented as vertical data-set of P/M ratios from cord blood, cord and five sections of placenta. Individuals were ranked based on the degree of scatter (intra-individual variation) observed.

Although preferential methylation of the presumed maternal allele was observed in almost all individuals (Figure 3), the distribution of paternal/maternal (P/M) methylation ratios at the *IGF2R* DMR in cord blood and cord showed that most individuals have an easily measurable level of methylation at the CpG within the *NotI* cleavage site on the presumed paternal allele (P/M>0.1, Figure 3), as has also been observed previously in peripheral blood from the CEPH families [28].

In cord blood, only 34% of the total population has low levels of methylation on the paternal allele (P/M allele ratios of <0.1) while a very small fraction of individuals (2%) have P/M allelic methylation ratios greater than 0.5. The distribution of individual P/M methylation ratios in cord (Figure 3) also showed a similar pattern to what was observed in cord blood. However, results from five placenta sections taken from the same individuals showed nearly complete loss of the methylation imprint (*i.e*. P/M ratios close to 1) at this locus in samples from multiple individuals in both *in vitro* and *in vivo* groups (Figure 3).

We found no difference in mean P/M ratios in cord blood, cord or placenta between the *in vitro* and *in vivo* groups, either comparing individual tissue types or combining all samples (Table 4). Cord blood allele-specific methylation ratio variance was greater in the *in vitro* group (*P* = 0.0016) but we did not attempt to calculate a cord blood stem cell number comparison because of the small number of samples on which to model the distribution. There was no significant difference in the population variance in the *in vitro* group in cord or placenta, although the presence of a substantial fraction of samples in both groups for which nearly complete loss of the methylation imprint (P/M>0.9) was observed is likely to affect our ability to distinguish such a difference.

**Table 4:** Intra- and inter-individual variation in DNA methylation at the *IGF2R* DMR.

Tissue	Group^a^	Mean	Variance	Means^b^ (*P*)	Variances (*P*)
Cord Blood	*In vitro*	0.1896	0.0305	0.6548	0.0016
	*In vivo*	0.1401	0.0085		
Cord	*In vitro*	0.1803	0.0257	0.5716	0.3072
	*In vivo*	0.1866	0.0210		
Placenta	*In vitro*	0.6199	0.0656	0.9782	0.4463
	*In vivo*	0.6263	0.0640		
Total	*In vitro*	0.5086	0.0918	0.6828	0.4483
	*In vivo*	0.4960	0.0936		

^**a**^P/M methylation ratios measured in cord blood, cord, and placenta tissue (5 sections) from 28 informative *in vitro* individuals and 27 informative *in vivo* individuals.

^**b**^Wilcoxon Rank Sums Test (*P*≤0.05 considered significant).

### IGF2, H19, and IGF2R Steady-State mRNA Levels

**Figure 4:**
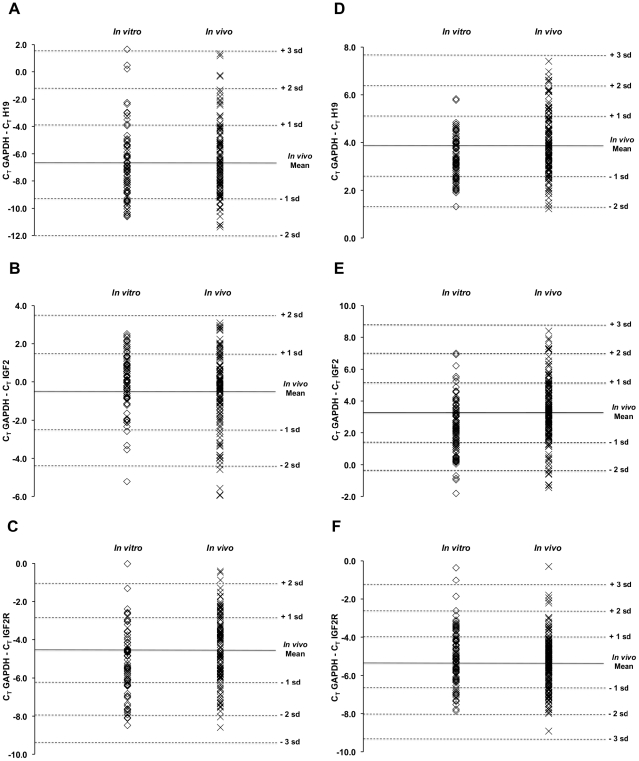
Scatter plots showing mRNA transcript levels in the *in vitro* and *in vivo* populations. Each symbol represents the mRNA level in one individual. (A) H19 in cord blood (*in vitro* n = 73, *in vivo* n = 118, fold change 0.88, *P* = 0.8774), (B) IGF2 in cord blood (*in vitro* n = 77, *in vivo* n = 116, fold change 1.03, *P* = 0.5551), (C) IGF2R in cord blood (*in vitro* n = 75, *in vivo* n = 121, fold change 0.61, *P* = 0.0039), (D) H19 in placenta (*in vitro* n = 84, *in vivo* n = 135, fold change 0.72, *P* = 0.0193), (E) IGF2 in placenta (*in vitro* n = 98, *in vivo* n = 160, fold change 0.52, *P*<0.0001), and (F) IGF2R in placenta (*in vitro* n = 97, *in vivo* n = 148, fold change 1.18, *P* = 0.2227).

We measured steady-state IGF2, H19 and IGF2R mRNA levels in cord blood and placenta from children conceived *in vitro* or *in vivo* (Table 5, Figure 4). In addition to the children who were informative for allele specific DMR methylation (Figure 1 and Figure 3), we also measured mRNA levels in the children who were not informative. Mean cord blood IGF2R mRNA levels were significantly lower in the *in vitro* group (fold change  = 0.61, *P = *0.0039). Mean placental IGF2 and H19 mRNA levels were also significantly lower in the *in vitro* group (fold change  = 0.52, *P*<0.0001, and fold change  = 0.72, *P* = 0.0193, respectively).

**Table 5:** Differences in transcript level between *in vitro* and *in vivo* groups.

Tissue	Gene	No. individuals (*in vitro*/*in vivo*)	Fold change^a^	*P* b
Cord Blood	H19	73/118	0.88	0.8774
	IGF2	77/116	1.03	0.5551
	IGF2R	75/121	0.61	0.0039
Placenta	H19	84/135	0.72	0.0193
	IGF2	98/160	0.52	<0.0001
	IGF2R	97/148	1.18	0.2227

^**a**^Fold change in *in vitro* conceived individuals compared with *in vivo* conceived individuals, obtained using the ΔΔC_T_ method [62].

^**b**^Student's T-test (*P*≤0.05 considered significant).

## Discussion

We have examined intra- and inter-individual variation in DNA methylation at the differentially methylated regions (DMRs) of the *IGF2/H19* and *IGF2R* loci in cord blood, cord and five sections of placenta from a population of children conceived *in vitro* or *in vivo*. Although a significant fraction of individuals in both groups do appear to maintain the *IGF2*/*H19* methylation imprint “correctly”, with M/P ratios of <0.1 (93.6% of peripheral blood samples are below this M/P ratio, Sandovici et al., 2003) [28], we found substantial intra-individual and inter-individual variation in allele-specific methylation in both groups in all three tissues: 8/46 individuals in the *in vivo* group and 7/40 individuals in the *in vitro* group have cord blood M/P ratios above this level (n.b. of the 56 informative *in vivo* and 45 informative *in vitro* individuals shown in Figure 1, cord blood samples were unavailable for 10 of the *in vivo* and five of the *in vitro* children). The acquisition of CpG methylation on only a fraction of maternal *IGF2*/*H19* DMRs in so many individuals suggests that there is extensive population level variation in the time at which methylation imprints become set during development, especially in extraembryonic lineages. This assertion receives further support from the analysis of intra-individual variation in P/M methylation ratios at an *IGF2R* DMR. Very few individuals (one in the *in vivo* group, two in the *in vitro* group) maintained this imprint “correctly” in all samples (even if the threshold for “correctly” is reduced to P/M ratios <0.2) and the discrepancy between maintaining the imprint in embryonic and extraembryonic tissue is even more pronounced (Figure 3). The observation of greater epigenetic variation in extraembryonic than in embryonic tissues is consistent with observations at a number of imprinted loci, including *IGF2*/*H19*, in the mouse [11]. We believe this is the first time this observation has been made in the human. Parental allele-specific mean methylation ratios at the *IGF2/H19* DMR were greater in the *in vitro* group, indicating acquisition of methylation on maternal alleles. This finding is consistent with observations made at *Igf2*/*H19* in the mouse [23].

The total variance in M/P ratio at *IGF2*/*H19* was significantly greater in cord blood and cord from the *in vitro* group, with a suggestive *P-*value (0.0620) for placenta. These findings indicate an association between ART and the magnitude of the variance in parental allele-specific methylation patterns. The mechanism by which greater variance might be created in the *in vitro* group is unclear but could be related to the number of trophoblast stem cells that give rise to the placenta in each group. If fewer trophoblast stem cells give rise to the placenta in the *in vitro* group, one expects greater intra-individual variation in somatically heritable epigenetic marks if the population of stem cells contains cells with more than one epigenetic state (*i.e*. *IGF2*/*H19* DMRs that are methylated only on the paternal allele in some cells and methylated on both maternal and paternal alleles in others).

As an independent test of this prediction, we measured X chromosome inactivation ratios in five sections of placenta in females from the *in vitro* (50 individuals) and *in vivo* groups (54 individuals) in order to estimate the number of trophoblast stem cells that give rise to the placenta in each group. The closest fit to the *in vitro* X-inactivation distribution corresponds to nine trophoblast stem cells while the closest fit to the *in vivo* distribution corresponds to 11 trophoblast stem cells. We additionally estimated the number of trophoblast stem cells in each group by comparing the distribution of M/P *IGF2*/*H19* DMR methylation ratios from the two comparison groups. The closest fit to the distribution of M/P ratios in placenta in children conceived *in vitro* corresponds to eight trophoblast stem cells and the closest fit in children conceived *in vivo* corresponds to 10 trophoblast stem cells. Overall, these estimates of between group intra-individual epigenetic variation are consistent with the prediction that overall greater variance in M/P *IGF2*/*H19* DMR methylation ratios in the *in vitro* group is associated with fewer trophoblast stem cells.

Although our statistical estimates of the number of trophoblast stem cells is imprecise, it gives one some confidence that similar absolute numbers are obtained (Table 2) from estimates of epigenetic variance at two different loci, examining different individuals with each assay. Whether the calculated difference in number of trophoblast stem cells reflects designation of trophoblast stem cells at an earlier stage of development (when fewer cells are present in the embryos) or whether embryos from the *in vitro* group have fewer cells than *in vivo* embryos at comparable stages cannot be determined from these data, however, previous reports suggest that *in vitro* mouse embryos may contain fewer cells than *in vivo* embryos at the same developmental time [41]. Our approach of using epigenetic variance to calculate the number of trophoblast stem cells is, for obvious reasons, the only opportunity for estimating this number in the human because direct comparisons of cell numbers in *in vitro* and *in vivo* embryos is not possible.

We note that while we have uncovered locus-specific differences in the level of epigenetic variation in children conceived *in vitro*, we cannot distinguish whether the differences are due to some aspect of the assisted reproduction process or is related to the underlying infertility. In fact, the characteristic of epigenetic variance, itself, may be under genetic control and may also be influenced by the environment [42]. Greater variance in trait value, even without changes in trait mean, is predicted to have a substantial positive effect on fitness in a changing environment [42]. In this regard, we note that *in vitro* conception is associated with at least two changes of environment (hormonal stimulation, retrieval of ova from the maternal environment to fertilization and culture *in vitro*, followed by return to the maternal environment). A larger-scale epigenetic screen is required in order to determine whether there is a tendency for *in vitro* conception to be associated with overall increased variance of epigenetic marks.

Several observations are noteworthy about the steady-state mRNA levels measured for IGF2 in placenta. First, IGF2 mRNA levels in placentas from *in vitro* conceived children, as a group, were approximately half of what was observed in children conceived *in vivo*. This observation is consistent with experiments demonstrating reduced Igf2 mRNA levels in placentas from mouse embryos subject to *in vitro* manipulations [20]. Second, reduction in IGF2 mRNA levels in the human placentas does not occur in conjunction with loss of methylation at the paternal DMR, as expected if transcript levels are controlled by genomic imprinting alone. Furthermore, we did not observe that increased levels of methylation at the maternal DMR induced a coordinate level of transcription from the maternal *IGF2* allele. In fact, given that IGF2 transcript levels vary by more than an order of magnitude between individuals (Figure 4) and almost by that much between samples within a single placenta (Figure S1), the mechanism by which natural selection might act in a population, on a process whose postulated design is to reduce transcription by half (from two alleles to one) is unclear. Along these same lines, we observed no correlation between birth weight and IGF2 transcript levels in either placenta or cord blood, whether or not birth weights were corrected for gestational age (Figure S2). This last observation was not completely unexpected, as several laboratories have failed to observe a correlation between IGF2 mRNA levels and birth weight [43]–[45]. In this regard, it is likely that epigenetic marking of genes according to parental origin plays an important role in other processes associated with reproduction and the formation of gametes, such as chromosome pairing and recombination [46]–[49]. The selective force for the maintenance of imprinting in these processes is both direct (successful recombination is required for successful gametogenesis) and related to reproductive success.

We also observed that mean steady-state levels of IGF2R mRNA were lower in cord blood from the *in vitro* group. This locus does not appear to be transcriptionally imprinted in most humans [40], [50], although the preferential methylation of only one parent's allele (the maternal) is conserved [26], [27]. Although not transcriptionally imprinted, we did note an inverse correlation between methylation of the paternal allele and overall transcript level, indicating that “aberrant” methylation of the “incorrect”, paternal allele does have a small effect (accounting for ∼10% of the variance, Figure S3). Lower IGF2R mRNA level in the *in vitro* group is, on the face of it, in contrast to expectations. If IGF2R is a receptor that acts as a “sink” for IGF2 [51], [52], one might expect children conceived *in vitro* to have higher levels of IGF2R because they have a higher probability of low birth weight [53]. In any case, we did not find any correlation between birth-weight, IGF2 levels, IGF2R levels or IGF2/IGF2R ratios (Figure S2).

Overall, our results indicate that epigenetic modifications at *IGF2*/*H19* and *IGF2R* are subject to frequent changes during early development, especially in extraembryonic tissues. Although not all of the epigenetic changes appear to be manifested as significant differences in DNA methylation, conception *in vitro* is associated with gene expression differences for all three genes in some tissues. Whether the gene expression differences between *in vitro* and *in vivo* groups are also a manifestation of what appears to be a smaller number of trophoblast stem cells in children from the *in vitro* group is a subject for future investigation.

## Materials and Methods

### Subjects

The cases/*in vitro* group are newborns conceived by assisted reproductive technology at a single infertility treatment center so that the clinical and laboratory procedures are uniform. The parents of the control/*in vivo* group had no prior history of infertility and the index pregnancy was achieved without medical assistance, such as the use of infertility medications or treatments. All the *in vitro* patients were stimulated with commercially available gonadotrophin preparations. The embryo culture media and the incubation parameters were all the same. The cases and controls were matched with regards to maternal age, race and gestational age (Table S1). Written, informed consent was obtained in advance from the mother of each newborn (University of Pennsylvania I.R.B. approved protocol no. 804530). A summary of the assays used, number of individuals studied and the tissues investigated is provided in Table S2.

### Sample Collection

Cord blood, cord and placenta samples were collected from each *in vitro* and *in vivo* newborn. All cord blood samples were collected within 20 min of delivery. Tissue samples were stored at 4°C after delivery, and samples were collected within five hours of delivery [54]. The umbilical cord was wiped with normal saline and the cord vein was punctured with a 21G needle. Whole cord blood (6–10 ml) was collected in lavender topped vacutainer tubes at room temperature. The sample was shaken thoroughly to prevent clotting as the tube contains EDTA, ethylenediaminetetraacetic acid. An aliquot (3–4 ml) of cord blood was transferred to a 15 ml Falcon tube containing RNALater RNA Stabilization Reagent (Ambion, USA), following the manufacturers guidelines, to stabilize the RNA. The remaining cord blood in the lavender topped vacutainer tubes was saved for blood DNA extraction. All cord blood DNA and RNA samples were initially stored at 4°C, and nucleic acid extractions were performed within 2–4 days of collection.

Placental tissue (1.5–2.5 cm^3^) was excised from the fetal surface of the placenta and rinsed extensively with sterile saline solution to minimize maternal blood contamination. Each placenta was sampled from four quadrants and from directly behind the cord insertion site (this sample was used for the RT-PCR and pyrosequencing assays, as well as for the allele-specific methylation assays). A segment of umbilical cord (2 cm) was cut and treated in a similar fashion. Placental and cord tissue for RNA extraction were chopped into small pieces (0.5 cm^3^) and immersed in RNALater RNA Stabilization Reagent (Ambion, USA), following the manufacturers guidelines, as soon as possible after collection. All tissue DNA and RNA samples were initially stored at 4°C, and tissue digestion and nucleic acid extractions were performed within 2–4 days of collection. Approximately 4–5 mg of tissue was used for the DNA and RNA extraction procedures, and the remaining tissue was stored at −80°C.

### DNA and RNA Isolation

Cord blood DNA was isolated using the ArchivePure DNA Blood Kit (Fisher Scientific Company, USA) following the manufacturers guidelines. Tissue genomic DNA was extracted using standard phenol-chloroform extraction methods. The isolated DNA was dissolved in 10 mM TrisCl, pH 8.0, quantified using a spectrophotometer and stored at −80°C until further use. Cord blood RNA was isolated using the PerfectPure RNA Blood Kit (Fisher Scientific Company, USA) following the manufacturers guidelines.

Total cellular RNA was extracted from each tissue sample using TRIzol Reagent (Invitrogen Corporation, USA), according to the manufacturers instructions. The isolated RNA was dissolved in Milli-Q water, quantified using a spectrophotometer and stored at −80°C until further use.

### Parental Allele-Specific Methylation at *IGF2/H19* and *IGF2R* DMRs

There are many DMRs on chromosome 11, but the most consistent observations indicating a role in the control of transcription of the *IGF2* and *H19* genes involve a CpG island located in a 5 kb region centromeric to the *H19* gene, known as the *IGF2/H19* DMR [25]. CpG sites within this DMR on the paternal allele are normally methylated, while those on the maternal allele are normally unmethylated [30], [55]–[58]. This region also contains seven different binding sites for the CTCF protein [38] and the methylation status of the sixth binding site was found to be most consistently associated with the transcriptional status of both *IGF2* and *H19* [24].

The upstream *H19* sequence used in this study is available from GenBank (accession number AF125183). Allele-specific methylation was investigated by screening the DNA samples for a C/T polymorphism recognized by *CfoI* at the *IGF2/H19* DMR (near the sixth binding site for CTCF) [24]. After identifying maternal and paternal alleles of heterozygous individuals, a methylation-sensitive restriction endonuclease (*MluI*) was used to determine the methylation status of specific CpG sites within the DMR. If all paternal alleles are methylated and all maternal alleles are unmethylated at these sites in a sample of genomic DNA, then all maternal alleles should be cleaved by *MluI* while all paternal alleles will remain uncleaved. Amplification of the region by PCR using primers that flank the *MluI* site should amplify only paternal alleles (identified by post-PCR cleavage with *CfoI*). Amplification of maternal alleles indicates resistance to cleavage by *MluI*. This may occur as a result of methylation of the CpG site within the *MluI* recognition sequence (the principle upon which the assay is based), mutation of the *MluI* site or technical artifact. The latter two possibilities may be distinguished from the first by DNA sequencing, assay reproducibility and use of additional methylation-sensitive restriction endonucleases.

The *IGF2R* DMR is located in the second intron of *IGF2R* and is normally methylated on the maternal allele. The sequence of *IGF2R* is available from GenBank (accession number AF069333). Allele-specific methylation at the *IGF2R* DMR was investigated by screening the DNA for a C/T polymorphism recognized by *MspI*. After identifying maternal and paternal alleles of heterozygous individuals, a methylation-sensitive restriction endonuclease (*NotI*) was used to determine the methylation status of specific CpG sites within the DMR.

Genomic DNA (100 ng) from informative individuals was digested overnight at 37°C with an excess of a methyl-sensitive restriction endonuclease: *MluI* and *NotI* for the *IGF2/H19* and *IGF2R* DMRs, respectively. Control individuals who were homozygous for C alleles and homozygous for T alleles were also analyzed in each experiment.

After digestion, the enzymes were denatured and the digested DNA was amplified in a hot-stop PCR assay using the following primers: IGF2-F 5′-GAGATGGGAGGAGATACTAGG-3′ and IGF2-R 5′-GTCAGTTCAGTAAAAGGCTGG-3′ for the *IGF2/H19* DMR, and IGF2R-F 5′-GGCCGAGGCCTGGCATGTTGG -3′ and IGF2R-R 5′-TGGGGAAGCGCGAGAGGCCTAGG-3′ for the *IGF2R* DMR. After 30 cycles at 94°C for 30 s, 50°C for 30 s (*IGF2/H19*) or 63°C for 30 s (*IGF2R*), and 72°C for 1 min, we added 3 µCi α-^32^P dCTP for one additional cycle and a final elongation step (72°C for 7 min). PCR products were then digested overnight at 37°C with the enzyme used for identifying the parental origin of the alleles (*CfoI* for *IGF2/H19* and *MspI* for *IGF2R*). The samples were separated on denaturing 5% polyacrylamide gels and the intensity of the bands (alleles) were quantified using a PhosphoImage Reader FLA 5000 (FUJIFILM Medical Systems USA, Inc.).

### Analyses of *IGF2*/*H19* DMR Methylation by Pyrosequencing

We used a custom pyrosequencing assay for the *IGF2/H19* DMR (NCBI36:11,2019856-2019740) which included five CpGs. Genomic DNA (500 ng per sample) was bisulfite treated using EZ Gold DNA Methylation Kit (Zymo Research, USA) following the manufacturers protocol.

Bisulfite treated DNA was used for generating PCR amplified templates for pyrosequencing. The PCR primer sequences were: forward 5′- GGGGTTATTTGGGAATAGG-3′ and biotin labeled reverse, 5′- CCAAACCATAACACTAAAACCCTC-3′. The PCR reaction (30 µl) was following: 25 ng of bisulfite DNA, 0.75 U HotStar Taq Polymerase (Qiagen, USA), 1X PCR buffer, 3 mM MgCl_2_, 200 µM of each dNTPs, 6 pmol forward primer and 6 pmol reverse primer. Recommended PCR cycling conditions were: 95°C for 15 min; 45 cycles (95°C for 30 s; 60°C for 30 s; 72°C for 30 s); 72°C for 5 min. The biotinylated PCR product (10 µl) was used for each sequencing assay with the following sequencing primer: 5′- GAATAGGATATTTATAGGAG-3′. Pyrosequencing was done using the PSQ96HS system according to standard procedures using Pyro Gold Reagent kits (Biotage, Sweden). Methylation was quantified using Pyro Q-CpG Software (Biotage, Sweden), which calculates the ratio of converted C's (T's) to unconverted C's at each CpG and expresses this as a percentage methylation.

### Quantitative Real-Time RT-PCR

First-strand cDNA was obtained using SuperScript III Reverse Transcriptase (RT) (Invitrogen Corporation, USA). To produce cDNA from total RNA, a mixture containing 0.5–1 µg extracted total RNA, 0.5 µg oligo(dT)18 primer and 1 µl dNTP mix (10 mM each) in a final 13 µl reaction volume was heated to 65°C for 5 min, cooled down on ice for 1 min, and then added to a 7 µl reaction mixture containing 4 µl SuperScript III RT buffer (10), 1 µl DTT (0.1 M), 1 µl RNaseOUT Recombinant RNase inhibitor (40 U/µl; Invitrogen Corporation, USA) and 1 µl SuperScript III M-MLV reverse transcriptase (200 U/µl). The samples were mixed and incubated at 50°C for 60 min. Reactions were terminated at 70°C for 15 min and the RT products were stored at −20°C until further use.

Quantitative real-time RT-PCR assays were carried out using a 7700 Sequence Detector (Applied Biosystems, USA). GAPDH, which has previously been used as a housekeeping gene in placenta by several investigators [59]–[61], was used as the housekeeping gene. All the placental tissue samples were from the third trimester. There was a positive correlation between GAPDH expression and the expression of another commonly used housekeeping gene HPRT, when studied in the same samples (Figure S4).

Steady-state mRNA levels of IGF2, H19, IGF2R and housekeeping gene GAPDH were measured using gene-specific primers and QuantiFast SYBR Green PCR Master Mix (Qiagen, USA). The primer sequences were following: IGF2 Forward 5′-TCTGACCTCCGTGCCTA-3′, IGF2 Reverse 5′-TTGGGATTGCAAGCGTTA-3′, H19 Forward 5′-AGAAGCGGGTCTGTTTCTTTA-3′, H19 Reverse 5′-TGGGTAGCACCATTTCTTTCA-3′, IGF2R Forward 5′-ACCTCAGCCGTGTGTCCTCT-3′, IGF2R Reverse 5′-CTCCTCTCCTTCTTGTAGAGCAA-3′, GAPDH Forward 5′-GAGTCAACGGATTTGGTCGT-3′, and GAPDH Reverse 5′-TTGATTTTGGAGGGATCTCG-3′. PCR reactions were performed by mixing 1 µl of cDNA (50 ng/µl placenta, 25 ng/µl cord blood) with 24 µl of reaction mixture (12.5 µl QuantiFast SYBR Green PCR Master Mix (2X), 2.5 µl forward primer (10 µM), 2.5 µl reverse primer (10 µM), and 6.5 µl nuclease free dH2O) and amplified under the following conditions: 95°C for 5 min, followed by 40 cycles of 95°C for 10 s and 60°C for 30 s. A melting curve analysis of the PCR products was performed to verify their specificity and identity. PCR products were also run on 2% agarose gels to confirm the size of the amplified products. Relative gene expression levels were obtained using the ΔΔC_T_ method [62].

### Quantitative Analysis of *IGF2* and *H19* Imprinting Status

To avoid genomic DNA contamination during imprinting analysis, PCR was done across an intron-exon boundary and the cDNA products were gel-purified. The primers used for assaying *IGF2* imprinting were: primer 1, 5′−ATCGTTGAGGAGTGCTGTTTC−3′; primer 2, 5′−CGGGGATGCATAAAGTATGAG−3′; primer 3, 5′−CTTGGACTTTGAGTCAAATTGG−3′; and primer 4, 5′−GGTCGTGCCAATTACATTTCA−3′ [32]. Heterozygosity of an *ApaI* polymorphism in exon 9 of *IGF2* was ascertained by doing PCR with genomic DNA using primers 3 and 4 across the *ApaI* site, and the PCR product was digested with *ApaI*. Imprinting status was ascertained by doing RT-PCR, using primers 1 and 2 in exons 8 and 9, respectively. The cDNA PCR product, which is shorter than any possible contaminating genomic DNA product because of intron splicing, was electrophoresed and purified from a 2% agarose gel using the QIAquick Gel Extraction Kit (Qiagen, USA) following the manufacturers protocol. Hot-stop PCR was then done using primers 3 and 4, with α-^32^P dCTP added before the last cycle. The PCR product was digested with *ApaI* and then separated on denaturing 5% polyacrylamide gels and the intensity of the bands (alleles) were quantified using a PhosphoImage Reader FLA 5000 (FUJIFILM Medical Systems USA, Inc.).

The primers used for assaying *H19* imprinting were: *H19*-1, 5′-GGAGTTGTGGAGACGGCCTTGAGT-3′; *H19*-2, 5′-CCAGTCACCCGGCCCAGATGGAG-3′; and *H19*-3, 5′-CTTTACAACCACTGCACTACCTGAC-3′. Heterozygosity of an *RsaI* polymorphism in exon 5 of *H19* was ascertained by doing PCR with genomic DNA using primers *H19*-1 and *H19*-2 across the *RsaI* site, and the PCR product was digested with *RsaI*. Imprinting status was ascertained by doing RT-PCR, using primers *H19-*3 and *H19*-2 in exons 4 and 5, respectively. The cDNA PCR product, which is shorter than any possible contaminating genomic DNA product because of intron splicing, was electrophoresed and purified from a 2% agarose gel using the QIAquick Gel Extraction Kit (Qiagen, USA) following the manufacturers protocol. Hot-stop PCR was then done using primers *H19*-1 and *H19*-2, with α-^32^P dCTP added before the last cycle. The PCR product was digested with *RsaI* and then separated on denaturing 5% polyacrylamide gels and the intensity of the bands (alleles) were quantified using a PhosphoImage Reader FLA 5000 (FUJIFILM Medical Systems USA, Inc.).

### X-Inactivation Assay

X-chromosome inactivation ratios were assayed using previously published modifications of a methylation-sensitive PCR assay [33], [36], [63]–[65]. We measured the methylation status of a CpG site that is correlated with the expression of alleles at the X-linked, highly polymorphic androgen receptor (*AR*) locus. Genomic DNA from cord blood, cord and five sections of placenta was available for 50 *in vitro* and 54 *in vivo* females who were heterozygous for *AR* alleles that differed by more than one CAG repeat.

Genomic DNA from females who were heterozygous (informative) at the highly polymorphic (CAG)n repeat of the X-linked AR gene was amplified after previous overnight digestion with *HhaI* methyl-sensitive restriction endonuclease, with primers (AR1: 5′-AGAGGCCGCGAGCGCAGCAC-3′ and AR2: 5′-ACTCCAGGGCCGACTGCGGC-3′), which flank the repeat and two *HhaI* sites. We added a radiolabeled nucleotide for the last cycle of ‘hot-stop’ PCR, rather than a single end-labeled primer, to increase the signal. After 27 cycles at 94°C for 1 min, 68°C for 1 min, and 72°C for 1 min, 3 µCi α-^32^P dCTP was added for one additional cycle. PCR products were separated on denaturing 5% polyacrylamide gels and the intensity of the alleles was quantified by using the PhosphoImage Reader FLA 5000 (FUJIFILM Medical Systems USA, Inc.) [65]. As a way of quantifying the degree of skewing, *i.e.,* the degree to which the somatic cells of an individual female deviated from a 1∶1 ratio, the intensity of the upper allele divided by the sum of the intensities of both alleles was computed for each individual.

### Statistical Analysis

The statistical significance of the methylation datasets representing the *in vivo* and *in vitro* group was examined using the Wilcoxon Rank Sums Test. Data from the real time RT-PCR experiments were analyzed using Student's T-test. The number of cells was estimated using the method described by Amos-Landgraf et al. (2006) and Mclaren A (1972) [34], [35]. *P-*values ≤0.05 were considered significant.

## Supporting Information

Figure S1 Intra-individual variation in IGF2 mRNA expression. Steady-state IGF2 mRNA levels measured in five sections of placenta in 12 individuals. IGF2 transcript levels vary by more than an order of magnitude (*n.b.:* each unit on the vertical axis is a power of 2) between individuals and between samples within some individuals.
(0.05 MB TIF)

Figure S2 Lack of correlation between birth weight percentiles and IGF2 or IGF2R mRNA levels in cord blood and placenta. Birth weight percentiles in the *in vitro* and *in vivo* populations plotted *versus* cord blood transcript levels of (A) IGF2, (B) IGF2R, (C) IGF2/IGF2R, and placenta transcript levels of (D) IGF2, (E) IGF2R, and (F) IGF2/IGF2R. Although average birth weight was lower in the *in vitro* group, we found little correlation between birth weight and IGF2 or IGF2R transcript levels (regression lines are shown, straight and dashed lines for *in vivo* and *in vitro* populations, respectively, and the maximum r^2^of 0.2433 is found in (B), even when birth weights were corrected for gestational age [Min, et al; Oken, et al; Yarkoni, et al]. These findings are consistent with previous observations from studies in human IUGR placentae [43]. [Min SJ, Luke B, Min L, Misiunas R, Nugent C, et al. (2004) Birth weight references for triplets. Am J Obstet Gynecol 191(3): 809-814. Oken E, Kleinman KP, Rich-Edwards J, Gillman MW (2003) A nearly continuous measure of birth weight for gestational age using a United States national reference. BMC Pediatr 8(3): 6. Yarkoni S, Reece EA, Holford T, O'Connor TZ, Hobbins JC (1987) Estimated fetal weight in the evaluation of growth in twin gestations: a prospective longitudinal study. Obstet Gynecol 69(4): 636-639.)
(0.46 MB TIF)

Figure S3 Correlation between IGF2R expression and P/M methylation ratios in cord blood.
(0.06 MB TIF)

Figure S4 Positive correlation between GAPDH expression and the expression of another commonly used housekeeping gene, HPRT, when studied in the same placenta samples.
(0.12 MB TIF)

Table S1 Patient demographics.
(0.04 MB DOC)

Table S2 Summary of assays used, number of individuals studied, and tissues investigated.
(0.05 MB DOC)
