# Supplementary figures and images for: Inter- and Intra-Individual Variation in Allele-Specific DNA Methylation and Gene Expression in Children Conceived using Assisted Reproductive Technology
Source: PLoS Genet. 2010 Jul 22;6(7):e1001033. doi: 10.1371/journal.pgen.1001033 (PMC2908687; doi:10.1371/journal.pgen.1001033)

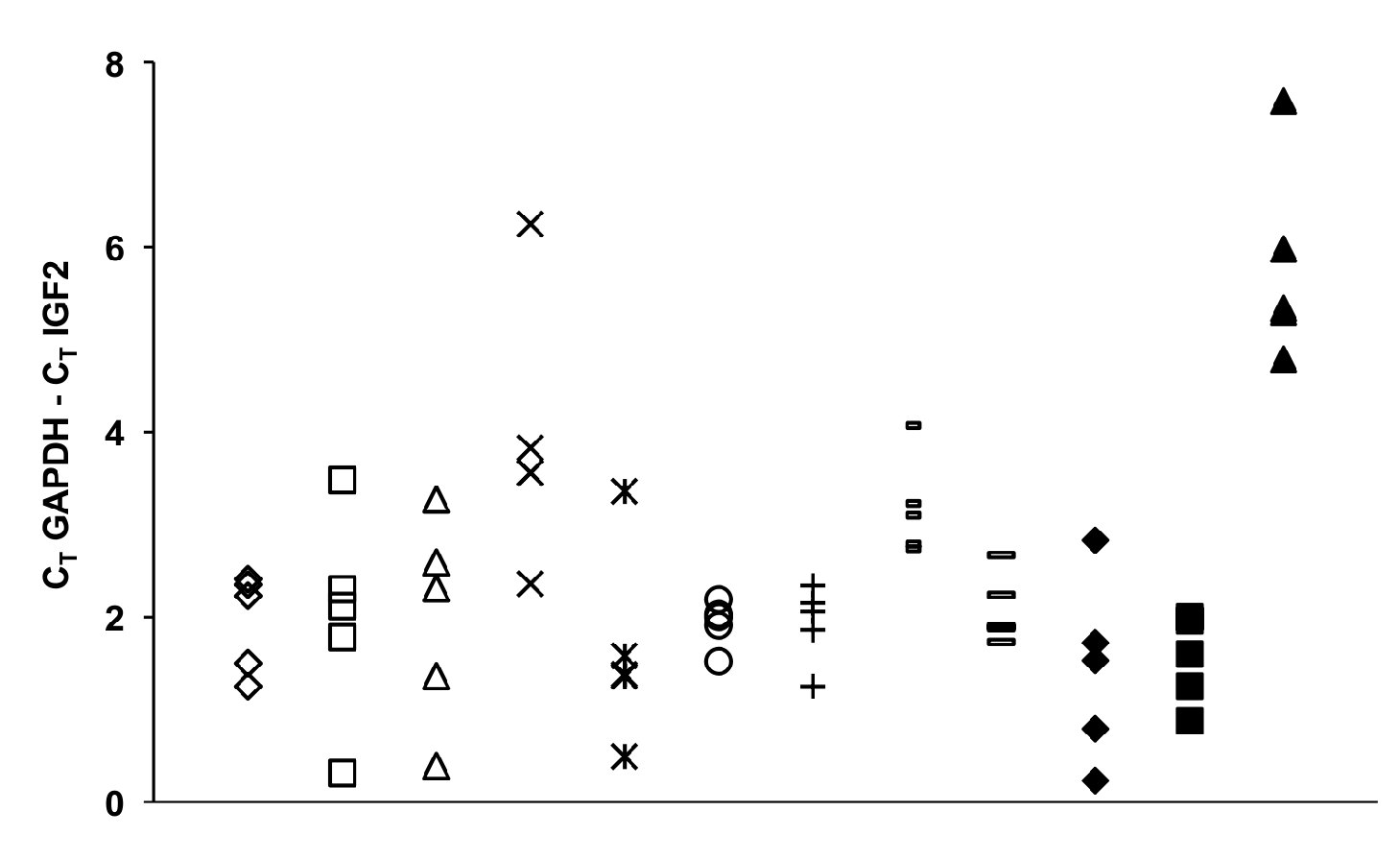

Supplement: Figure S1 — Intra-individual variation in IGF2 mRNA expression. Steady-state IGF2 mRNA levels measured in five sections of placenta in 12 individuals. IGF2 transcript levels vary by more than an order of magnitude (n.b.: each unit on the vertical axis is a power of 2) between individuals and between samples within some individuals. (0.05 MB TIF) [file pgen.1001033.s001.tif]

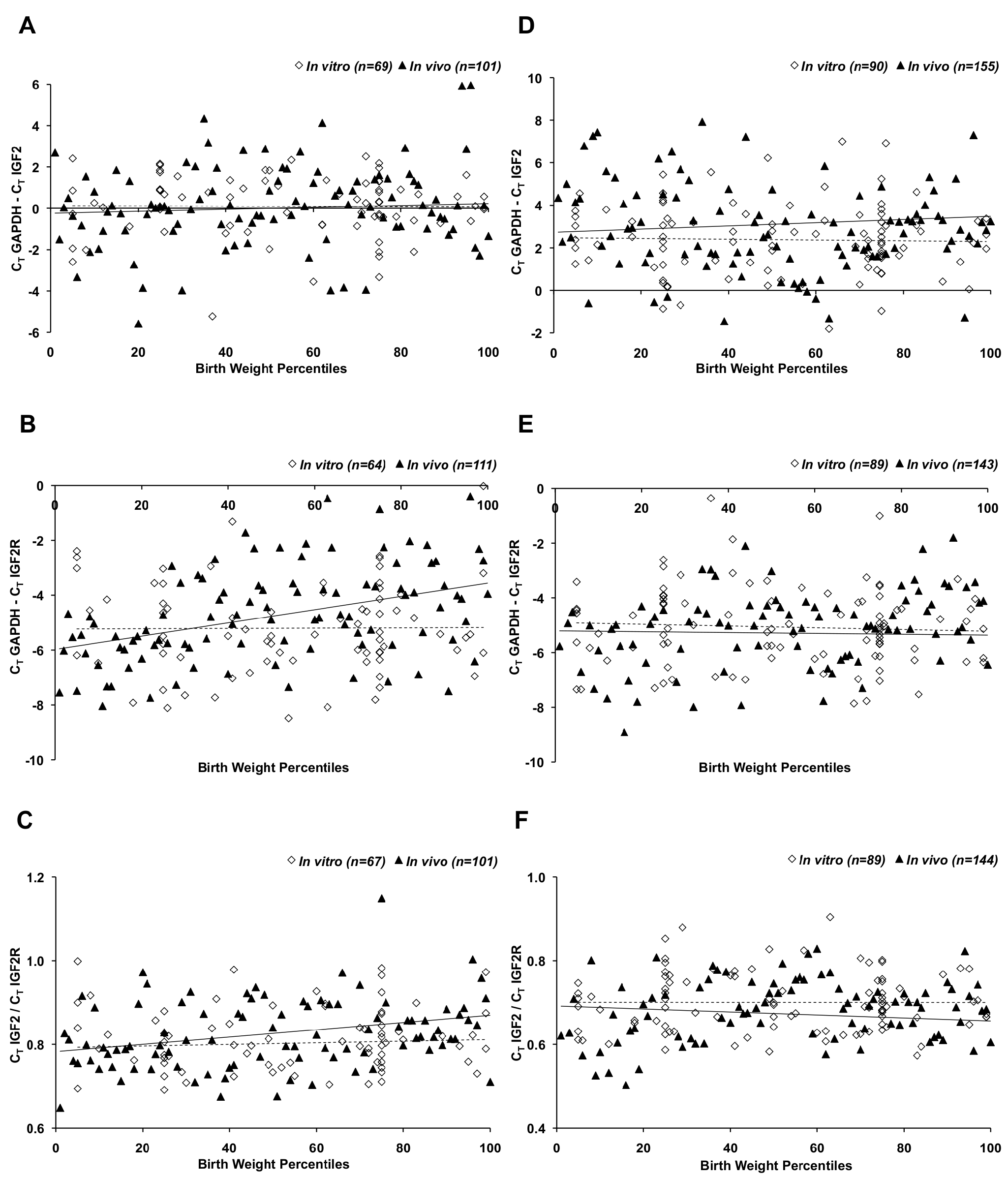

Supplement: Figure S2 — Lack of correlation between birth weight percentiles and IGF2 or IGF2R mRNA levels in cord blood and placenta. Birth weight percentiles in the in vitro and in vivo populations plotted versus cord blood transcript levels of (A) IGF2, (B) IGF2R, (C) IGF2/IGF2R, and placenta transcript levels of (D) IGF2, (E) IGF2R, and (F) IGF2/IGF2R. Although average birth weight was lower in the in vitro group, we found little correlation between birth weight and IGF2 or IGF2R transcript levels (regression lines are shown, straight and dashed lines for in vivo and in vitro populations, respectively, and the maximum r2of 0.2433 is found in (B), even when birth weights were corrected for gestational age [Min, et al; Oken, et al; Yarkoni, et al]. These findings are consistent with previous observations from studies in human IUGR placentae [43]. [Min SJ, Luke B, Min L, Misiunas R, Nugent C, et al. (2004) Birth weight references for triplets. Am J Obstet Gynecol 191(3): 809-814. Oken E, Kleinman KP, Rich-Edwards J, Gillman MW (2003) A nearly continuous measure of birth weight for gestational age using a United States national reference. BMC Pediatr 8(3): 6. Yarkoni S, Reece EA, Holford T, O'Connor TZ, Hobbins JC (1987) Estimated fetal weight in the evaluation of growth in twin gestations: a prospective longitudinal study. Obstet Gynecol 69(4): 636-639.) (0.46 MB TIF) [file pgen.1001033.s002.tif]

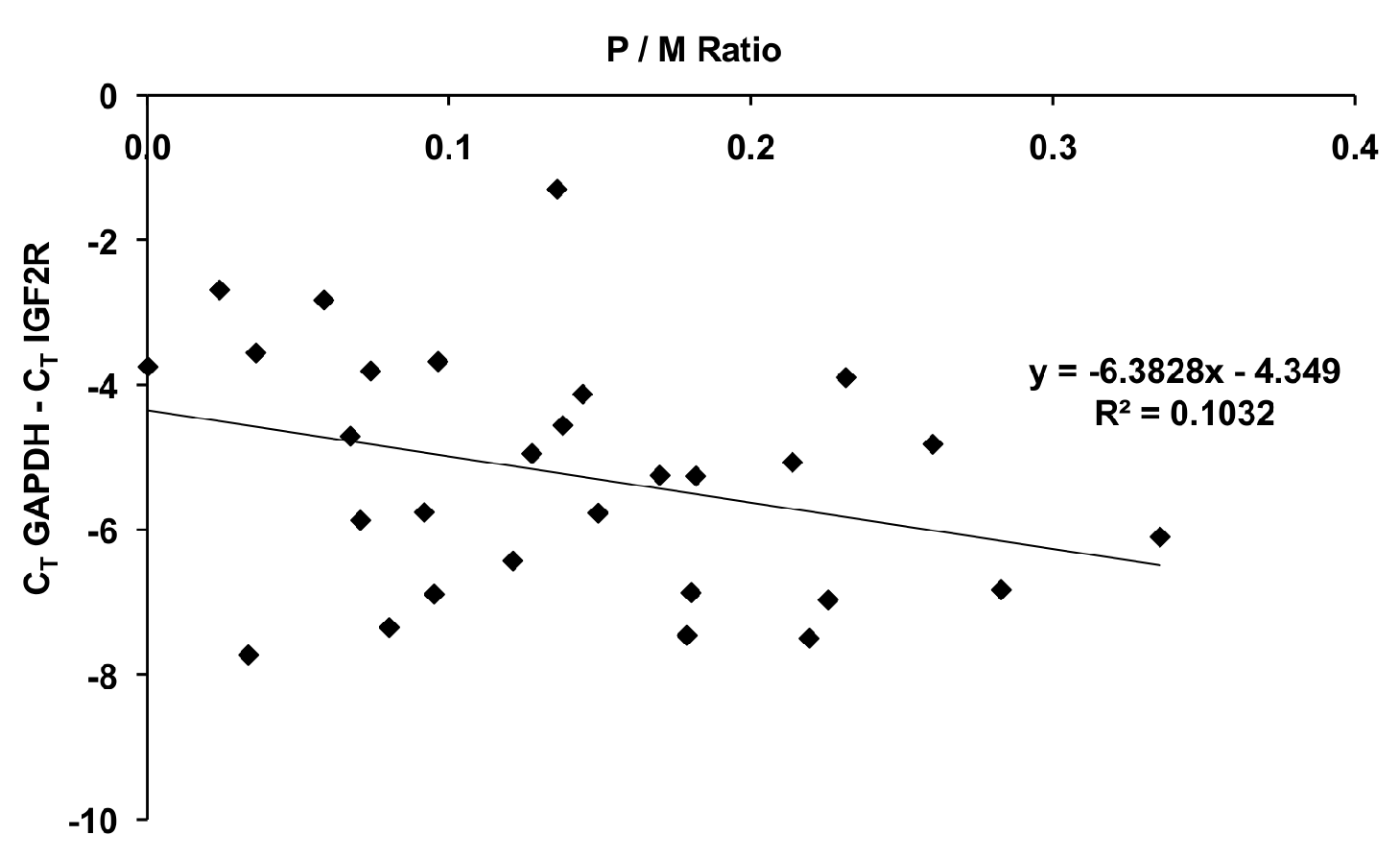

Supplement: Figure S3 — Correlation between IGF2R expression and P/M methylation ratios in cord blood. (0.06 MB TIF) [file pgen.1001033.s003.tif]

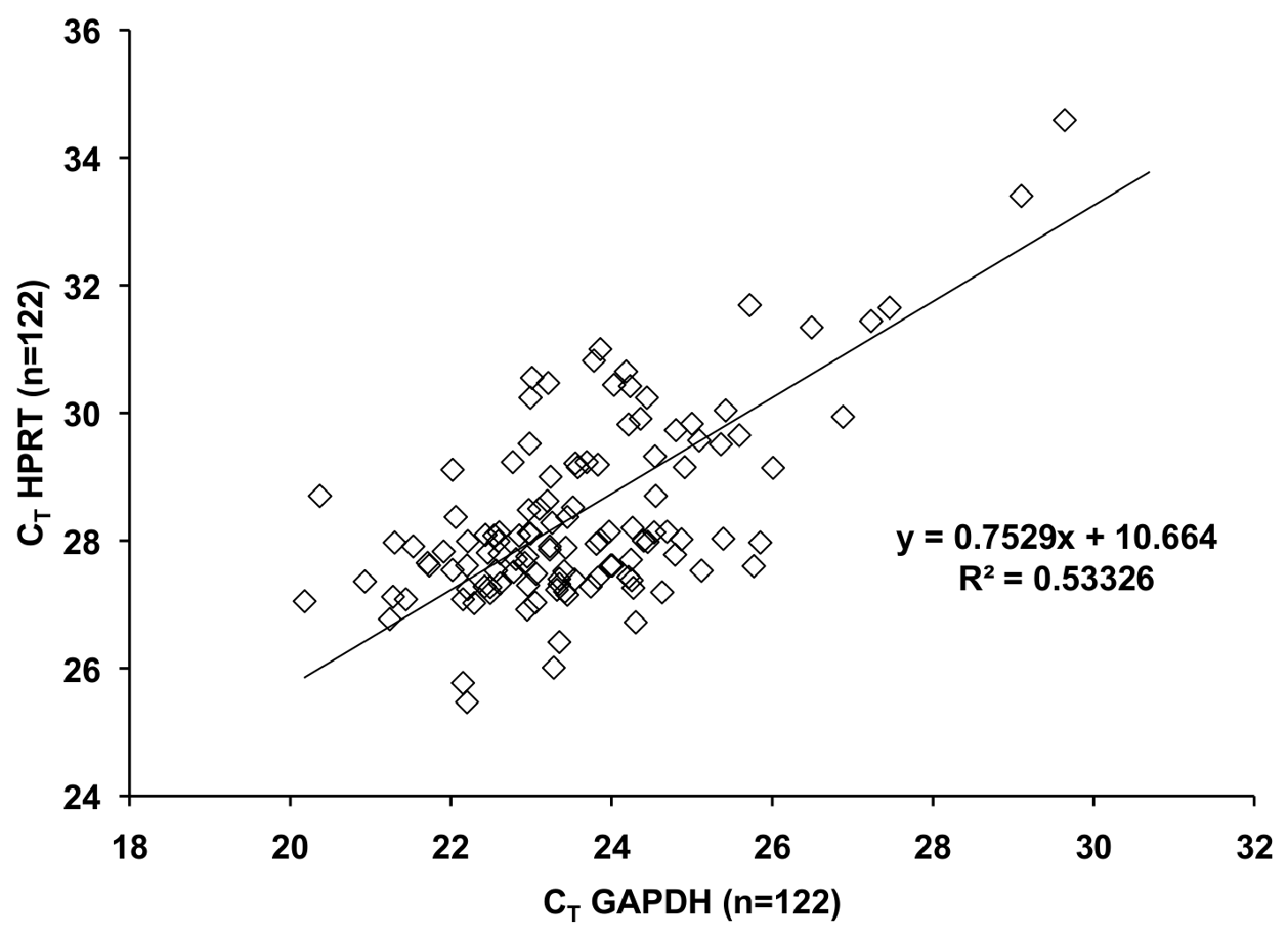

Supplement: Figure S4 — Positive correlation between GAPDH expression and the expression of another commonly used housekeeping gene, HPRT, when studied in the same placenta samples. (0.12 MB TIF) [file pgen.1001033.s004.tif]
