# Supplementary material for: Inter- and Intra-Individual Variation in Allele-Specific DNA Methylation and Gene Expression in Children Conceived using Assisted Reproductive Technology
Source: PLoS Genet. 2010 Jul 22;6(7):e1001033. doi: 10.1371/journal.pgen.1001033 (PMC2908687; doi:10.1371/journal.pgen.1001033)
Supplement: Table S1 — Patient demographics. (0.04 MB DOC) [file pgen.1001033.s005.doc]

**Table S1.** **Patient Demographics.**

| **Characteristics** | ***In vitro* (n=98)** | ***In vivo* (n=160)** |
| --- | --- | --- |
| Gestational age, wks (mean ± SD) | 38 ± 2.5 | 39 ± 1.2 |
| > 37 wks | 82 (84%) | 154 (96%) |
| 34 – 37 wks | 9 (9%) | 5 (3%) |
| 30 – 34 wks | 7 (7%) | 1 (1%) |
| Twin sets | 19 | 2 |
| Triplets | 1 | 0 |
| Newborns with low birth weight (<10^th^ percentile) | 9 (10%) | 17 (11%) |
| Male infants | 52% | 54% |
| Female infants | 48% | 46% |
| Maternal age, yrs (mean ± SD) | 36 ± 4 | 31 ± 6 |
