# Supplementary material for: Inter- and Intra-Individual Variation in Allele-Specific DNA Methylation and Gene Expression in Children Conceived using Assisted Reproductive Technology
Source: PLoS Genet. 2010 Jul 22;6(7):e1001033. doi: 10.1371/journal.pgen.1001033 (PMC2908687; doi:10.1371/journal.pgen.1001033)
Supplement: Table S2 — Summary of assays used, number of individuals studied, and tissues investigated. (0.05 MB DOC) [file pgen.1001033.s006.doc]

**Table S2.** **Summary of assays used, number of individuals studied and tissues investigated.**

| **Assay** | **Group** | **No. Individuals** | **Details** |
| --- | --- | --- | --- |
| *IGF2/H19* allele specific methylation | *In vitro* | 45^a^ | Cord blood, cord and five sections placenta analyzed for each individual. |
|  | *In vivo* | 56 ^a^ |  |
| *IGF2R* allele specific methylation | *In vitro* | 28^b^ | Cord blood, cord and five sections placenta analyzed for each individual. |
|  | *In vivo* | 27^b^ |  |
| *IGF2/H19* pyrosequencing | *In vitro* | 26 | One section placenta (from behind the cord) analyzed for each individual. |
|  | *In vivo* | 31 |  |
| X-inactivation | *In vitro* | 50^c^ | Five sections placenta analyzed for each individual. |
|  | *In vivo* | 54^c^ |  |
| RT-PCR | *In vitro* | 98 | Cord blood and one section placenta (from behind the cord) analyzed for each individual. |
|  | *In vivo* | 160 |  |

^a^ Number of informative individuals for C/T polymorphisms in *CfoI* site in *IGF2/H19* DMR.

^b^ Number of informative individuals for C/T polymorphisms in *MspI* site in *IGF2R* DMR.

^c^ Number of females informative at (CAG)n repeat in *AR* gene.
